# Wood-Based Panel Industry Wastewater Meets Microbial Fuel Cell Technology

**DOI:** 10.3390/ijerph17072369

**Published:** 2020-03-31

**Authors:** Renata Toczyłowska-Mamińska

**Affiliations:** Warsaw University of Life Sciences – SGGW, Institute of Biology, Department of Physics and Biophysics, 159 Nowoursynowska St., 02-776 Warsaw, Poland; renata_toczylowska@sggw.pl

**Keywords:** microbial fuel cell, wastewater, wood-based panels, plywood, wood hydrothermal treatment

## Abstract

Although the wood-based panel industry is not considered to be a water-consuming sector, it generates ca. 600 M m^3^ of wastewater every year on a global scale. The wastewater is usually highly polluted and environmentally toxic even after dilution. Common wastewater treatment techniques require high-energy input or addition of various chemicals to the treated wastewater, which cause secondary pollution and production of toxic sludge. Microbial fuel cells (MFCs) have become an attractive technology, allowing for zero-energy treatment of various types of wastewater with simultaneous production of electric current. Recent investigations have shown that MFCs can also be utilized for sustainable treatment and energy production from the wastewater generated by the wood-based panel industry. This article contains a critical summary of the investigations in this field as well as a discussion of the research needed and perspectives for the future.

## 1. Introduction

Sectors of the wood-based panel industry differ in terms of water usage in the production process. In contrast to pulp and paper production, which is known to be one of the largest industrial water consumers [1], wood-based panel production is commonly considered to be a dry sector with low water demand, whereas world wood-based panel production can generate up to ca. 600 M m^3^ of wastewater every year [2]. Wood panels are the most popular among wood-based materials, and during the production process water is used to soak logs for plywood production for cooling down materials and equipment or for cleaning the facilities. Wastewater from production of wood-based materials is usually highly polluted. Chemical oxygen demand (COD) values were measured in a wide range from 200 to 11,000 mg O_2_/L depending on the operation stage that generated the wastewater [3,4,5]. They contain a wide range of various substances among which wood degradation products, wood extractives, heavy metals or even surfactants introduced during cleaning processes can be found. Investigations indicate that wood industry wastewaters are of high environmental toxicity even after extensive dilution [6].

One of the most water-consuming processes during panel production is wood hydrothermal treatment during which logs are soaked in basins filled with water, which results in an increase in wood plasticity before debarking and veneer cutting. As hydrothermal treatment is usually carried out at temperatures of 50–70 °C, under such conditions cellulose, lignin and products of their degradation as well as wood extractives (e.g., resin acids, tannins or phenolic compounds) are eluted [7], so wastewater COD values are usually above 3000 mg O_2_/L (Table 1). Wood extractives are naturally produced in trees to protect them against pathogen attack (e.g., bacterial or fungal), but their presence in wastewater makes such waste environmentally toxic [8]. Beside extractives, lignin and its derivatives are also resistant to microbial degradation and cause toxic and hormonal effects in aquatic environments [9]. To limit fresh water consumption, many plants reduce the frequency of water exchange in basins, which leads to accumulation of contaminants and higher environmental toxicity. This in turn necessitates the use of effective in situ treatment procedures to reduce wastewater contamination.

Among the treatment techniques that are utilized for wastewater from the wood-based panel industry the most popular is coagulation using aluminum sulphate. The exemplary efficiency of COD removal from plywood wastewater was reported to be 57%, but was accompanied by so-called secondary pollution (introduction of chemicals to the wastewater during treatment) and generation of toxic sludge that needs to be managed [11]. Application of adsorption and coagulation methods allowed for 91% removal of lipophilic extractives from wet debarking process wastewater, though managing the sludge generated during treatment was still a necessity [12]. An example of a method that avoids wastewater secondary pollution is an advanced oxidation process known as the Fenton process, which when applied to sawmill wastewater treatment resulted in an 80% COD reduction [13]. Unfortunately, the application of advanced oxidation processes involves high treatment costs and a high-energy demand that can reach 11 kWh/kg COD [1].

Investigations conducted by many research groups around the world have indicated that the problem of sustainable wastewater treatment may be resolved by the application of microbial fuel cell (MFC) technology [14]. The undeniable supremacy of MFCs over other wastewater treatment techniques is due to (1) no energy input, (2) no chemicals added during the process and (3) the electric current produced during treatment as an energy surplus. MFCs are bioelectrochemical systems that allow organic matter to be converted directly into electric current by utilizing microorganisms [15]. A typical MFC is composed of two electrodes, an anode and a cathode, placed in compartments separated by a cation-specific membrane. In the anode chamber, under anaerobic conditions, electrogenic microorganisms oxidize organic substances and transfer released electrons to the anode. Protons pass through the cation-specific membrane to the cathode [16]. Electrons are then directed from the anode to the cathode through an electric circuit comprising external resistance, which results in the generation of electric current in the system. In the cathode compartment, electrons and protons combine with oxygen, and water is formed. In the case of complex substrates, such as wastewater, microbial consortia need to be used. In such consortia there are fermentative and methanogenic species that decompose complex substances into simple organics that can be further used by electrogenic species for current production [17]. MFCs were successfully applied in the treatment of various types of wastewater (including industrial) with the use of dual-chamber and single-chamber reactors [18].

**Figure 1:**
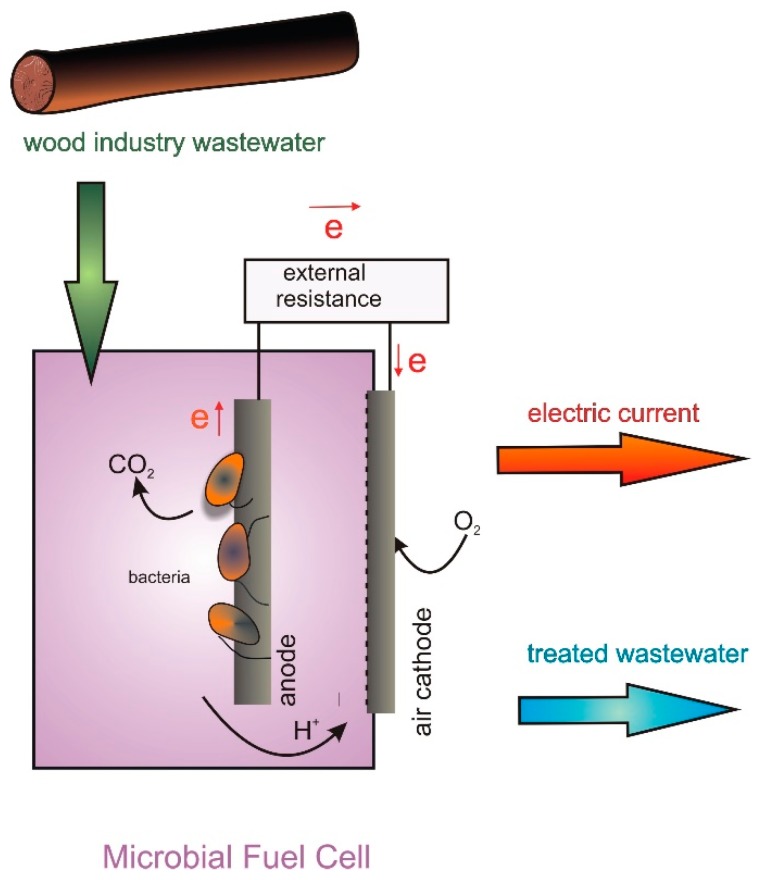
Schematic representation of the production of electric current from hydrothermal treatment wastewater (WHTW) in a single-chamber air-cathode microbial fuel cell (MFC) with simultaneous treatment of wastewater.

At the Warsaw University of Life Sciences, for the first time MFC technology has been applied in the management of wood industry wastewater. All the investigations were conducted with single-chamber reactors with an air cathode (Figure 1) [5]. In a single-chamber reactor a graphite anode was placed in an anaerobic chamber filled with hydrothermal treatment wastewater (WHTW). The microorganisms in the WHTW oxidized organic substances present in wastewater, and electrogenic species of the consortium transfer released electrons to the anode. The electrons were then transferred through an external circuit to the air cathode, which resulted in current generation in the system. In this way current was produced from WHTW in the MFC, and simultaneously wastewater was treated by the decomposition of organic matter, which caused the reduction of wastewater COD.

This article summarizes the research on the application of MFC technology for the treatment of WHTW generated from the wood-based panel industry with simultaneous current production. Critical factors affecting power production and wastewater treatment efficiency, as well as future research directions, are discussed.

## 2. Significance of External Resistance

External resistance is a basic parameter that influences wastewater treatment efficiency and power production in MFCs. It is known that maximum power is produced in an MFC when external resistance equals the internal resistance of the system [19]. However, it was recently reported that the effect of external resistance on wastewater treatment efficiency is different for various wastewater types [2]. When malodorous surface water or brackish wastewater were used in MFCs, COD removal efficiency was constant, independent of the resistance applied [20,21]. According to Katuri et al. [22], the highest COD removal was obtained for the highest applied resistance, and the current produced was maximum, when mixed brewery and domestic wastewaters were used as substrate.

Previous investigations on WHTW in single-chamber MFCs revealed that external resistance is an important parameter that strongly influences both wastewater treatment efficiency and power production. Changing the external resistance from 500 to 1000 Ω resulted in an increase in COD removal efficiency from 65% to 94% and an increase in power production from ca. 10 to ca. 100 mW/m^2^ [2]. Though 1000 Ω was the optimal resistance for maximizing COD removal efficiency, maximum power was not observed with the same resistance. Maximum power production (178 mW/m^2^; ca. 4 W/m^3^) was measured for R = 2000 Ω, at which COD removal efficiency was not maximal (ca. 80%). As different resistances are needed for the optimization of either treatment efficiency or power production, the selection of external resistance depends on the choice of the parameter to be maximized. Since wood-based industry wastewaters differ greatly in terms of degree of pollution and contaminant concentration, the choice of optimal external resistance should be made individually for every specific wastewater used.

## 3. Raw Wastewater Microbial Consortium Composition

Analysis of wastewater generated during wood hydrothermal treatment indicates that its microbial composition differs between plants or even between individual basins within a single plant. The phenomenon results from different conditions of wood treatment and different origins of logs. The most critical factors are the time for which the water is utilized in the basins (which can vary from one week to several months), but also temperature (usually in the range 50–65 °C), wood species type (hardwood or softwood) and microbial composition of the soil in which the trees were grown. Table 1 shows the chemical composition of wastewater from basins in different plants where hardwood was treated hydrothermally. When the water in the basin was changed once a year [4] or less frequently [5], the total amount of solids in the wastewater was much higher (> 2000 mg/L) than in the case of water changed every few weeks, where total solids were below 500 mg/L [2,10]. The amount of lignin and tannins was also higher (560–870 mg/L) when the water was changed less frequently versus 250 mg/L in water changed every few weeks [2,10]. However, the amount of chlorides and ammonium nitrogen in WHTW is strongly influenced by wood origin. Large differences in the concentrations of these ions (e.g., chlorides 560 and 37 mg/L) were measured in samples taken from the same basin at intervals of a few months, after the treatment of different hardwood species [4].

**Figure 2:**
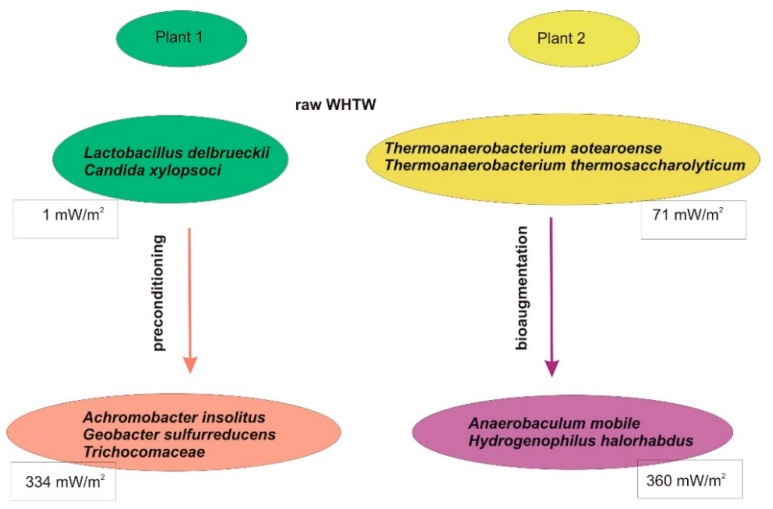
The composition of microbial consortia in WHTW from different plants.

Investigations into power production in MFCs fed WHTW sampled from various plants demonstrated that power is not always produced from this substrate, despite similar COD values. Analysis of microorganisms by genomic sequencing in various samples of WHTW revealed apparent differences in microbial consortia composition (Figure 2). The presence of different species in wastewater consortia resulted in different power production in MFCs. In plant 1 where water in the basin was changed every few weeks, the dominant genera were *Lactobacillus* and *Candida* (Figure 2). Both *L. delbrueckii* and *C. xylopsoci* are species that assimilate simple sugars, but they are not able to use the cellulose and lignin that are abundant in WHTW. Thus, power production from raw WHTW sampled from plant 1 was not possible, as the measured values did not exceed 1 mW/m^2^. In plant 2, WHTW was sampled from the basin where water had not been changed for 4 years, and only the evaporated portion was replenished. As a result, the microbial consortium was dominated by *Thermoanaerobacterium* species, which produced a power density of 71 mW/m^2^ in the MFC from raw WHTW without any supplementation. *Thermoanaerobacterium* are thermophilic bacteria known for their ability to ferment cellulose and hemicellulose with end products such as acetate or hydrogen that can be used by electrogenic bacteria for current production [5].

## 4. Wastewater Preconditioning

Wood-industry wastewaters are difficult substrates for microorganisms to utilize. The presence of cellulose and lignin derivatives, as well as wood extractives toxic to bacteria, make such environments unfavorable for microorganism development. However, bacteria are known for their remarkable adaptation abilities, which enable them to survive in harsh conditions. Preconditioning of the consortium to give a more favorable environment for microorganisms may help desirable species to acclimatize and develop. Anaerobic sludge consortium preconditioning with nitroethane was previously found to cause a four-fold increase in power density that was related to suppression of methanogenic bacteria development [23]. A two-fold increase in power produced in MFCs inoculated with anaerobic sludge was also observed after inoculum temperature pretreatment [24].

Investigations into WHTW showed that power production in WHTW-fed MFCs can be enhanced by bioaugmentation of WHTW by the addition of municipal wastewater [5]. This approach allowed development of new consortia where the most abundant microorganisms were *Anaerobaculum mobile* and *Hydrogenophilus halorhabdus*, and the power produced was increased from 71 to 360 mW/m^2^ (Figure 2, plant 2). In this consortium *H. halorhabdus* was a thermophilic species with the ability to decompose complex substrates, while *A. mobile* was previously described as a sulfur reducer, which explains its electrogenic activity [5]. Similarly, preconditioning of WHTW from plant 1 (Figure 2) at 45 °C allowed for development of a new consortium dominated by fungi of *Trichocomaceae spp*. together with the bacteria species *A. insolitus* and *Geobacter sulfurreducens*. In this consortium, *Trichocomaceae spp*. decomposed cellulose and fermented it to acids and alcohols that were further used by *A. insolitus* to produce acetic acid, a substrate for electrogenic *G. sulfurreducens,* one of the best known current-producing species [10]. Development of such a consortium allowed for stimulation of power production of 334 mW/m^2^ (ca. 8 W/m^3^), while no power was produced without preconditioning.

## 5. Conclusions

Previous investigations into WHTW indicate that the application of MFC technology leads to a reduction in water usage and prevents the generation of environmentally toxic wastewater in the wood-based panel industry. MFCs surpass every available method of wastewater treatment as not only do they eliminate secondary wastewater pollution and energy consumption, they also allow simultaneous energy production during the treatment process. The maximum COD removal efficiency obtained to date is 94%, and the maximum power density is 9 W/m^3^, which corresponds to a current density of 18 A/m^3^. However, optimization of wastewater treatment or current production in MFCs should be run separately as different conditions are needed to maximize each of these two parameters.

**Figure 3:**
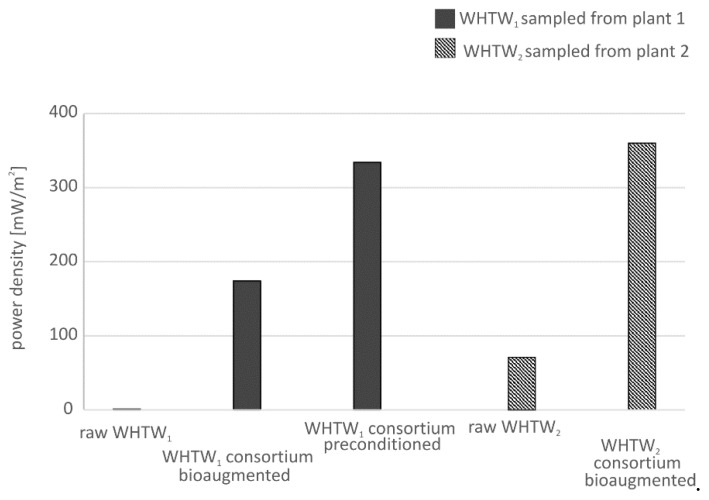
Summary of different power densities produced in MFCs fed WHTW.

The amount of power produced is strictly dependent on the external resistance applied, so the first step should be to choose the optimal resistance for maximizing power production. As shown recently by Kloch and Toczyłowska-Mamińska [2], changing external resistance in the range from 100 to 2000 Ω allowed power production to be enhanced from 8 to 178 mW/m^2^ during WHTW treatment in MFCs. One very important parameter that affects the amount of power produced is the microbial composition of consortia in raw wastewater. Our previous results proved that some WHTW (consortia dominated by *Lactobacillus spp*.) yielded no power production, while for WHTW sampled from another plant, power production of 71 mW/m^2^ was obtained [5]. This explains why optimization of current production in MFCs should match the specific wastewater used in situ. If a consortium that naturally occurs in wastewater does not result in satisfactory current production, it can be bioaugmented or preconditioned. In some cases, bioaugmentation of a consortium by mixing WHTW with municipal wastewater may result in a few-fold increase of power produced in MFCs [5]. In turn, temperature preconditioning of WHTW can be an effective method for stimulating power production from the WHTW that previously yielded no power production (Figure 3).

Investigations into optimization of WHTW treatment in MFCs revealed that WHTW treatment is dependent on external resistance and the time of MFC operation. Maximum COD removal efficiency was observed after 3 weeks of MFCs working at a resistance of R = 1000 Ω. Application of optimal resistance and MFC start-up time allowed for COD reduction to the value 200 mg O_2_/L, which is a sufficient level to recycle the treated water in the plant [2].

## 6. Perspectives

Future investigations dealing with the application of MFC technology for the treatment of wood-based panel industry wastewater assisted by current production should focus on maximizing power production in the system by researching the optimal conditions required for microorganisms to develop and produce electricity. The currently obtained maximum power production of 9 W/m^3^ is still not satisfactory, though when we take into account single-basin volume (ca. 2000 m^3^) and the fact that there can be several basins in a single plant, we can obtain power production as high as ca. 300 kW from WHTW generated in one plant. If MFC technology was also applied to the treatment of wastewater other than WHTW generated in the plant then the power produced would be even greater. Besides power production, there is a need for further investigation into wood-based panel industry wastewater treatment efficiency. Although high efficiency of COD removal has been obtained, there have been no studies that consider the removal of heavy metals or detailed investigations into removal of lignin, tannins, resin acids and the products of their degradation. The preliminary investigations performed at the Warsaw University of Life Sciences indicate that lignin is not removed in MFCs during the WHTW treatment process [10]. Thus, this should be a stimulus for detailed studies in this field aimed at researching the conditions that might resolve the issue of lignin.

Currently, on the world scale we are able to produce a maximum of ca. 5 GW of energy from wastewater generated during the production of wood-based panels with the use of MFC technology. This shows that MFCs have a large potential application in the wood industry, and the obtained power production is a good starting point for further investigations, especially when compared to the amount of energy produced by photovoltaics, which in Poland is ca. 1 GW a year [25]. Considering current production, we could have saved 600 M m^3^ of water a year if MFC technology had been put into practice in the wood-based panel industry.

## Figures and Tables

**Table 1:** Chemical composition of wastewater generated during wood hydrothermal treatment, sampled from different plants, in milligrams per liter (mg/L).

	Reference [4]	Reference [4]	Reference [4]	Reference [5]	Reference [2,10]
COD	5150	7860	6834	3343	4900
pH	4.8	4.8	5.1	5.2	5.6
Total solids	2550	n.a.	n.a.	2200	476
Total suspension	1800	n.a.	1780	480	500
Cl-	560	n.a.	37	50	37
Total Fe	n.a.	n.a.	n.a.	16	78
NH4-N	1.7	n.a.	50	2.2	n.a.
Phenols	52	n.a.	94	n.a.	n.a.
Lignin/tannins	560	870	n.a.	n.a.	250
SO_4_^2–^	160	n.a.	< d.l.	100	n.a.
Cellulose	n.a.	n.a.	n.a.	n.a.	280
Organic acids	n.a.	n.a.	n.a.	n.a.	Max. 62 for formic acid and 44 for acetic acid
Furfural	n.a.	n.a.	n.a.	n.a.	25
Alcohols	n.a.	n.a.	n.a.	n.a.	Max. 6 for 1-phenylethanol

## References

[1] Toczyłowska-Mamińska R. (2017). Limits and perspectives of pulp and paper industry wastewater treatment –A review. Renew Sustain. Energy Rev..

[2] Kloch M., Toczyłowska-Mamińska R. (2020). Toward optimization of wood industry wastewater treatment in microbial fuel cells—Mixed wastewaters approach. Energies.

[3] Rueda-Márquez J.J., Levchuk I., Uski J., Sillanpää M., Acevedo A., Manzano M.A. (2016). Post-treatment of plywood mill effluent by Multi-Barrier Treatment: A pilot-scale study. Chem. Eng. J..

[4] Klauson D., Klein K., Kivi A., Kattel E., Viisimaa M., Dulova N., Velling S., Trapido M., Tenno T. (2015). Combined methods for the treatment of a typical hardwood soaking basin wastewater from plywood industry. Int. J. Environ. Sci. Technol..

[5] Toczyłowska-Mamińska R., Szymona K., Kloch M. (2018). Bioelectricity production from wood hydrothermal-treatment wastewater: Enhanced power generation in MFC-fed mixed wastewaters. Sci. Total Environ..

[6] Laohaprapanon S., Kaczala F., Salomon P.S., Marques M., Hogland W. (2012). Wastewater generated during cleaning/washing procedures in a wood-floor industry: toxicity on the microalgae *Desmodesmus subspicatus*. Environ. Technol..

[7] Vek V., Oven P., Poljanšek I. (2016). Review on lipophilic and hydrophilic extractives in tissues of common beech. Drvna. Ind..

[8] Hedenström E., Fagerlund Edfeldt A., Edman M., Jonsson B.G. (2016). Resveratrol, piceatannol, and isorhapontigenin from Norway spruce (Picea abies) debarking wastewater as inhibitors on the growth of nine species of wood-decaying fungi. Wood Sci. Technol..

[9] Leiviskä T., Rämö J., Nurmesniemi H., Pöykiö R., Kuokkanen T. (2009). Size fractionation of wood extractives, lignin and trace elements in pulp and paper mill wastewater before and after biological treatment. Water Res..

[10] Sekrecka-Belniak A., Pielech-Przybylska K., Dziekońska-Kubczak U., Toczyłowska-Mamińska R. Stimulation of electricity production in microbial fuel cells via regulation of syntrophic consortium development. Appl. Energy.

[11] Jokela P., Keskitalo P. (1999). Plywood mill water system closure by dissolved air flotation treatment. Water Sci. Technol..

[12] Munoz M., Pliego G., de Pedro Z.M., Casas J.A., Rodriguez J.J. (2014). Application of intensified Fenton oxidation to the treatment of sawmill wastewater. Chemosphere.

[13] Leiviskä T., Sarpola A., Tanskanen J. (2012). Removal of lipophilic extractives from debarking wastewater by adsorption on kaolin or enhanced coagulation with chitosan and kaolin. Appl. Clay. Sci..

[14] Logan B.E. (2008). Microbial Fuel Cells.

[15] Mohan S.V., Velvizhi G., Modestra J.A., Srikanth S. (2014). Microbial fuel cell: Critical factors regulating bio-catalyzed electrochemical process and recent advancements. Renew Sustain. Energy Rev..

[16] Leong J.X., Daud W.R.W., Ghasemi M., Liew K.B., Ismail M. (2013). Ion exchange membranes as separators in microbial fuel cells for bioenergy conversion: A comprehensive review. Renew Sustain. Energy Rev..

[17] Toczyłowska-Mamińska R., Szymona K., Król P., Gliniewicz K., Pielech-Przybylska K., Kloch M., Logan B.E. (2018). Evolving microbial communities in cellulose-fed microbial fuel cell. Energies.

[18] Gude V.G. (2016). Wastewater treatment in microbial fuel cells—An overview. J. Clean. Prod..

[19] Kamau J.M., Mbui D.N., Mwaniki J.M., Mwaura F.B., Kamau G.N. (2017). Microbial fuel cells: influence of external resistors on power, current and power density. J. Thermodyn. Catal..

[20] Wang H., Fu B., Xi J., Hu H.Y., Liang P., Huang X., Zhang X. (2019). Remediation of simulated malodorous surface water by columnar air-cathode microbial fuel cells. Sci. Total Environ..

[21] Pham H.T., Vu P.H., Nguyen T.T.T., Bui H.V.T., Tran H.T.T., Tran H.M., Nguyen H.Q., Kim H.B. (2019). A laboratory-scale study of the applicability of a halophilic sediment bioelectrochemical system for in situ reclamation of water and sediment in brackish aquaculture ponds: Effects of operational conditions on performance. J. Microbiol. Biotechnol..

[22] Katuri K.P., Scott K., Head I.M., Picioreanu C., Curtis T.P. (2011). Microbial fuel cells meet with external resistance. Bioresour. Technol..

[23] Rajesh P.P., Noori M.T., Ghangrekar M.M. (2018). Pre-treatment of anodic inoculum with nitroethane to improve performance of a microbial fuel cell. Water Sci. Technol..

[24] Lu S., Xie B., Liu B., Lu B., Xing D. (2019). Neglected effects of inoculum preservation on the startup of psychrophilic bioelectrochemical systems and shaping bacterial communities at low temperature. Front. Microbiol..

[25] PV Magazine.com (2019). https://www.pv-magazine.com/2019/11/11/poland-tops-1-gw-of-solar/.

